# Crystal structure of 1,3-di­cyclo­hexyl-4,5-dimethyl-1*H*-imidazol-3-ium-2-carbodi­thio­ate chloro­form monosolvate

**DOI:** 10.1107/S1600536814023800

**Published:** 2014-11-05

**Authors:** Eyad Mallah, Kamal Sweidan, Wael Abu Dayyih, Manfred Steimann, Mahmoud Sunjuk

**Affiliations:** a Faculty of Pharmacy and Medical Science, University of Petra, Amman, Jordan; b Department of Chemistry, Faculty of Science, University of Jordan, Amman, Jordan; c Institut für Anorganische Chemie der Universität Tübingen, Auf der Morgenstelle 18, 72076 Tübingen, Germany; d Department of Chemistry, Faculty of Science, The Hashemite University, Jordan

**Keywords:** crystal structure, imidazole, carbodi­thio­ate, zwitterion

## Abstract

The title compound, C_18_H_28_N_2_S_2_·CHCl_3_, crystallizes as a zwitterion. The C—S bonds are almost equivalent, with lengths of 1.666 (3) and 1.657 (3) Å. The S—C—S bond angle is expanded to 129.54 (16)° and the N—C—N angle is reduced to the tetra­hedal value of 108.8 (2)°. In the crystal, adjacent mol­ecules are linked *via* C—H⋯S hydrogen bonds, forming chains along [100]. The chloro­form solvent mol­ecule, which is disordered over two positions [occupancy ratio = 0.51 (2):0.49 (2)], is linked to the chain by bifurcated C—H⋯(S,S) hydrogen bonds.

## Related literature   

For the properties and uses of heterocyclic carbenes, see: Kuhn & Al-Sheikh (2005 ▶); Kuhn *et al.* (1995 ▶, 1999 ▶); Mallah *et al.* (2009 ▶); Margulis & Tempelton (1962 ▶). For the structures of similar compounds, see: Winberg & Coffman (1965 ▶); Kuhn *et al.* (1994 ▶). For the synthesis of the starting material, see: Kuhn & Kratz (1993 ▶).
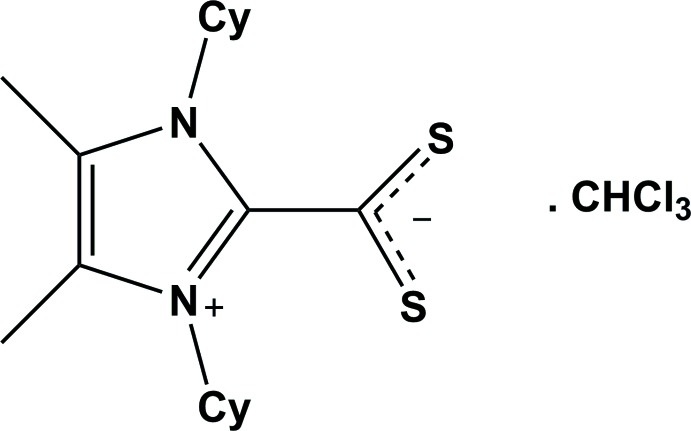


## Experimental   

### Crystal data  

C_18_H_28_N_2_S_2_·CHCl_3_
*M* *_r_* = 455.91
Monoclinic, 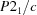

*a* = 8.4800 (17) Å
*b* = 16.227 (3) Å
*c* = 17.263 (4) Å
β = 98.78 (3)°
*V* = 2347.6 (8) Å^3^
*Z* = 4
Mo *K*α radiation
μ = 0.58 mm^−1^
*T* = 223 K
0.60 × 0.50 × 0.30 mm

### Data collection  

Enraf–Nonius CAD-4 diffractometer
Absorption correction: multi-scan (*CAD-4 Software*; Enraf–Nonius, 1998 ▶) *T*_min_ = 0.775, *T*_max_ = 0.939
5278 measured reflections
4797 independent reflections
3215 reflections with *I* > 2σ(*I*)
*R*_int_ = 0.062
3 standard reflections every 400 reflections intensity decay: 7%

### Refinement  

*R*[*F* ^2^ > 2σ(*F* ^2^)] = 0.053
*wR*(*F* ^2^) = 0.153
*S* = 1.04
4797 reflections
272 parameters
H-atom parameters constrained
Δρ_max_ = 0.40 e Å^−3^
Δρ_min_ = −0.45 e Å^−3^

### 

Data collection: *CAD-4 Software* (Enraf–Nonius, 1998 ▶); cell refinement: *CAD-4 Software*); data reduction: *HELENA*/*PLATON* (Spek, 2009 ▶); program(s) used to solve structure: *SHELXS97* (Sheldrick, 2008 ▶); program(s) used to refine structure: *SHELXL97* (Sheldrick, 2008 ▶); molecular graphics: *SHELXTL* (Sheldrick, 2008 ▶); software used to prepare material for publication: *SHELXTL*.

## Supplementary Material

Crystal structure: contains datablock(s) I, New_Global_Publ_Block. DOI: 10.1107/S1600536814023800/su5006sup1.cif

Structure factors: contains datablock(s) I. DOI: 10.1107/S1600536814023800/su5006Isup2.hkl

Supporting information file. DOI: 10.1107/S1600536814023800/su5006Isup3.cml

**Fig. 1.**
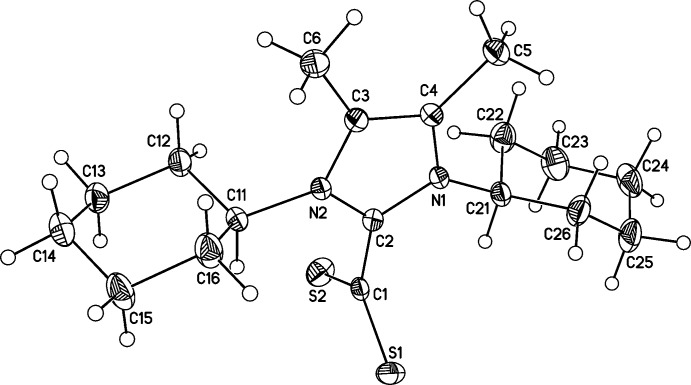
The molecular structure of the title molecule, with atom labelling. Displacement ellipsoids are drawn at the 20% probability level.

. DOI: 10.1107/S1600536814023800/su5006fig1.tif
The mol­ecular structure of the title mol­ecule, with atom labelling. Displacement ellipsoids are drawn at the 20% probability level.

CCDC reference: 1031415

Additional supporting information:  crystallographic information; 3D view; checkCIF report

## Figures and Tables

**Table 1:** Hydrogen-bond geometry (, )

*D*H*A*	*D*H	H*A*	*D* *A*	*D*H*A*
C15H15*B*S2^i^	0.98	2.76	3.650(4)	151
C30H30S1^i^	0.99	2.79	3.646(4)	145
C30H30S2^i^	0.99	2.68	3.543(3)	145

Symmetry code: (i) 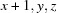.

## supplementary crystallographic information

### S1. Comment

Owing to their highly nucleophilic character, heterocyclic carbenes can act as organic ligands in complexes of metal and metalloid centers in a manner similar to the tertiary phosphanes (Kuhn *et al.*, 2005; Mallah *et al.*, 2009). The nucleophilic carbenes with carbon disulfide are known only sporadically to give disulfide adducts (Winberg *et al.*, 1965; Kuhn *et al.*, 1994). The formation of stable 1.3-dicyclohexyl-4.5-dimethylimidazol-2-ylidene adducts confirmed the previous approach about nucleophilic character of N-heterocyclic carbenes (Kuhn *et al.*, 1994.

The title compound, crystallized in the zwitterion form, Fig. 1. Bond length C1—C2 [1.484 (4) Å] is intermediate of carbon-carbon single and double bond lengths. The binding geometry of the CS_2_ [C1—S2 1.657 (3) Å, S2—C1—S1 129.54 (6)°] is similar to that in the structure of Et_3_PCS_2_ (Margulis & Tempelton, 1962) and are also very close to the bond lengths S1—C1, S2—C1 1.670 (5) Å] in IMCS_2_ (Kuhn *et al.*, 1999). Parallel to this, the expansion of the bond angle S1—C1—S2 [129.54 (6)°] and the reduction of the angle N2—C2—N1 [108.8 (3)°] were observed. A comparison of the structure data speaks for the extensive conservation the π-electrons configuration in the heterocyclic ring, so that the coordination of CS_2_ substantially the negative charge of C(2) to CS_2_ fragment delocalized.

The CS_2_ fragment is almost normal to the mean plane of the five-membered ring. The has the effect of isolating the two π systems which is also reflected in the relatively long C1—C2 bond, which is 1.484 (4) Å, confirms the sma behaviour in (Kuhn *et al.*, 1994). A comparison of the N-heterocyclic carbenes structures of (Kuhn *et al.*, 1995) and the crystal in the title compound, a marked expansion of the ring constant angle at the carbon-carbene by about 8° [N2—C2—N1 108.8 (2)°] as the only significant difference. The fact confirms the idea of a coordinative bond between the carbene system and the CS_2_-fragment without the participation of the respective π-systems.

In the crystal, adjacent molecules are linked *via* C-H···S hydrogen bonds forming chains along [100]. The chloroform molecule of solvent, which is disordered over two positions [occupancy ratio of 0.51 (2):0.49 (2)], is linked to the chain by bifurcated C-H···S,S hydrogen bonds (Table 1).

### S2. Experimental

The title compound was synthesized according to the published procedure (Kuhn & Kratz, 1993). 0.34 g (6.0 mmol) of CS_2_ was added to a solution of of 1.3-dicyclohexyl-4.5-dimethylimidazol-2-yliden (1.56 g, 6.0 mmol) in 20 ml of THF at 258 K. The reaction mixture was stirred over night and the precipitate formed was filtered off and dried *in vacuo*. Yield after recrystallization from methanol/diethylether was 1.72 g (85%), as red crystals.

### S3. Refinement

The C-bound H atoms were included in calculated positions and refined as riding: C- H = 0.97 - 0.99 Å with U_iso_(H) = 1.5U_eq_(C) for methyl H atoms and = 1.2U_eq_(C) for other H atoms. The chloroform molecule of solvent is disordered over two positions with an occupancy ratio of 0.51 (2):0.49 (2) .

### Crystal data

**Table:**

C_18_H_28_N_2_S_2_·CHCl_3_	*F*(000) = 960
*M**_r_* = 455.91	*D*_x_ = 1.290 Mg m^−^^3^
Monoclinic, *P*2_1_/*c*	Mo *K*α radiation, λ = 0.71073 Å
Hall symbol: -P 2ybc	Cell parameters from 25 reflections
*a* = 8.4800 (17) Å	θ = 7.6–13.7°
*b* = 16.227 (3) Å	µ = 0.58 mm^−^^1^
*c* = 17.263 (4) Å	*T* = 223 K
β = 98.78 (3)°	Block, red
*V* = 2347.6 (8) Å^3^	0.60 × 0.50 × 0.30 mm
*Z* = 4	

### Data collection

**Table:**

Enraf–Nonius CAD-4 diffractometer	3215 reflections with *I* > 2σ(*I*)
Radiation source: fine-focus sealed tube	*R*_int_ = 0.062
Graphite monochromator	θ_max_ = 26.4°, θ_min_ = 3.1°
ω scans	*h* = −10→10
Absorption correction: multi-scan (*CAD-4 Software*; Enraf–Nonius, 1998)	*k* = 0→20
*T*_min_ = 0.775, *T*_max_ = 0.939	*l* = −1→21
5278 measured reflections	3 standard reflections every 400 reflections
4797 independent reflections	intensity decay: 7%

### Refinement

**Table:**

Refinement on *F*^2^	Secondary atom site location: difference Fourier map
Least-squares matrix: full	Hydrogen site location: inferred from neighbouring sites
*R*[*F*^2^ > 2σ(*F*^2^)] = 0.053	H-atom parameters constrained
*wR*(*F*^2^) = 0.153	*w* = 1/[σ^2^(*F*_o_^2^) + (0.0736*P*)^2^ + 1.0555*P*] where *P* = (*F*_o_^2^ + 2*F*_c_^2^)/3
*S* = 1.04	(Δ/σ)_max_ < 0.001
4797 reflections	Δρ_max_ = 0.40 e Å^−^^3^
272 parameters	Δρ_min_ = −0.45 e Å^−^^3^
0 restraints	Extinction correction: *SHELXL97* (Sheldrick, 2008), Fc^*^=kFc[1+0.001xFc^2^λ^3^/sin(2θ)]^-1/4^
Primary atom site location: structure-invariant direct methods	Extinction coefficient: 0.0056 (12)

### Special details

**Table:**

Geometry. All e.s.d.'s (except the e.s.d. in the dihedral angle between two l.s. planes) are estimated using the full covariance matrix. The cell e.s.d.'s are taken into account individually in the estimation of e.s.d.'s in distances, angles and torsion angles; correlations between e.s.d.'s in cell parameters are only used when they are defined by crystal symmetry. An approximate (isotropic) treatment of cell e.s.d.'s is used for estimating e.s.d.'s involving l.s. planes.
Refinement. Refinement of *F*^2^ against ALL reflections. The weighted *R*-factor *wR* and goodness of fit *S* are based on *F*^2^, conventional *R*-factors *R* are based on *F*, with *F* set to zero for negative *F*^2^. The threshold expression of *F*^2^ > σ(*F*^2^) is used only for calculating *R*-factors(gt) *etc*. and is not relevant to the choice of reflections for refinement. *R*-factors based on *F*^2^ are statistically about twice as large as those based on *F*, and *R*- factors based on ALL data will be even larger.

### Fractional atomic coordinates and isotropic or equivalent isotropic displacement parameters (Å^2^)

**Table:**

	*x*	*y*	*z*	*U*_iso_*/*U*_eq_	Occ. (<1)
S1	0.33013 (10)	0.35829 (5)	0.21958 (5)	0.0524 (2)	
S2	0.08543 (11)	0.22805 (5)	0.16626 (5)	0.0601 (3)	
N1	0.2665 (3)	0.22155 (13)	0.36574 (12)	0.0374 (5)	
N2	0.4181 (3)	0.15925 (13)	0.29338 (12)	0.0357 (5)	
C1	0.2388 (3)	0.26807 (16)	0.22524 (14)	0.0366 (6)	
C2	0.3059 (3)	0.21643 (15)	0.29333 (14)	0.0346 (6)	
C3	0.4543 (3)	0.12642 (17)	0.36899 (15)	0.0401 (6)	
C4	0.3592 (3)	0.16530 (17)	0.41371 (15)	0.0410 (6)	
C5	0.3515 (5)	0.1536 (2)	0.49892 (17)	0.0598 (9)	
H5A	0.4239	0.1099	0.5195	0.090*	
H5B	0.2435	0.1390	0.5057	0.090*	
H5C	0.3821	0.2044	0.5269	0.090*	
C6	0.5769 (4)	0.0621 (2)	0.39258 (19)	0.0584 (9)	
H6A	0.5829	0.0508	0.4481	0.088*	
H6B	0.6798	0.0815	0.3821	0.088*	
H6C	0.5481	0.0121	0.3629	0.088*	
C11	0.4780 (3)	0.13436 (17)	0.22026 (15)	0.0401 (6)	
H11	0.4262	0.1722	0.1791	0.048*	
C12	0.4225 (4)	0.04907 (19)	0.19417 (18)	0.0486 (7)	
H12A	0.3061	0.0460	0.1898	0.058*	
H12B	0.4686	0.0087	0.2334	0.058*	
C13	0.4722 (4)	0.0282 (2)	0.11543 (19)	0.0596 (9)	
H13A	0.4464	−0.0296	0.1028	0.072*	
H13B	0.4113	0.0624	0.0746	0.072*	
C14	0.6454 (4)	0.0417 (2)	0.1153 (2)	0.0643 (9)	
H14A	0.6701	0.0321	0.0624	0.077*	
H14B	0.7064	0.0019	0.1507	0.077*	
C15	0.6956 (4)	0.1279 (3)	0.1410 (2)	0.0710 (11)	
H15A	0.6420	0.1677	0.1030	0.085*	
H15B	0.8109	0.1338	0.1422	0.085*	
C16	0.6529 (4)	0.1466 (2)	0.2229 (2)	0.0627 (9)	
H16A	0.7120	0.1096	0.2618	0.075*	
H16B	0.6818	0.2035	0.2378	0.075*	
C21	0.1355 (3)	0.27565 (18)	0.38309 (16)	0.0433 (7)	
H21	0.1026	0.3082	0.3348	0.052*	
C22	−0.0096 (4)	0.2273 (2)	0.3958 (2)	0.0629 (9)	
H22A	0.0144	0.1942	0.4437	0.075*	
H22B	−0.0408	0.1898	0.3516	0.075*	
C23	−0.1469 (4)	0.2874 (3)	0.4031 (2)	0.0765 (11)	
H23A	−0.1805	0.3142	0.3525	0.092*	
H23B	−0.2381	0.2564	0.4167	0.092*	
C24	−0.0988 (5)	0.3520 (3)	0.4644 (2)	0.0739 (11)	
H24A	−0.1858	0.3919	0.4634	0.089*	
H24B	−0.0820	0.3258	0.5162	0.089*	
C25	0.0500 (5)	0.3964 (2)	0.4524 (2)	0.0744 (11)	
H25A	0.0810	0.4343	0.4963	0.089*	
H25B	0.0285	0.4293	0.4043	0.089*	
C26	0.1874 (4)	0.33744 (19)	0.4464 (2)	0.0589 (9)	
H26A	0.2799	0.3682	0.4342	0.071*	
H26B	0.2184	0.3091	0.4965	0.071*	
C30	0.9203 (4)	0.42877 (19)	0.1636 (2)	0.034 (3)	0.51 (2)
H30	1.0037	0.3867	0.1791	0.041*	0.51 (2)
Cl1	0.9615 (8)	0.5094 (6)	0.2138 (5)	0.124 (3)	0.51 (2)
Cl2	0.9264 (10)	0.4611 (3)	0.0639 (4)	0.0889 (17)	0.51 (2)
Cl3	0.7332 (7)	0.3868 (5)	0.1695 (6)	0.091 (2)	0.51 (2)
C31	0.9117 (16)	0.4311 (8)	0.1563 (8)	0.095 (6)	0.49 (2)
H31	1.0026	0.3925	0.1682	0.113*	0.49 (2)
Cl1A	0.9542 (5)	0.5234 (3)	0.2235 (2)	0.0516 (14)	0.49 (2)
Cl2A	0.8855 (18)	0.4481 (8)	0.0636 (4)	0.161 (4)	0.49 (2)
Cl3A	0.7452 (9)	0.3845 (4)	0.1871 (7)	0.0886 (19)	0.49 (2)

### Atomic displacement parameters (Å^2^)

**Table:**

	*U*^11^	*U*^22^	*U*^33^	*U*^12^	*U*^13^	*U*^23^
S1	0.0600 (5)	0.0409 (4)	0.0582 (5)	−0.0029 (4)	0.0153 (4)	0.0107 (3)
S2	0.0651 (5)	0.0540 (5)	0.0536 (5)	0.0008 (4)	−0.0152 (4)	−0.0048 (4)
N1	0.0485 (13)	0.0363 (12)	0.0289 (11)	0.0043 (10)	0.0104 (9)	−0.0007 (9)
N2	0.0438 (12)	0.0333 (11)	0.0319 (11)	0.0045 (10)	0.0124 (9)	0.0009 (9)
C1	0.0458 (15)	0.0362 (14)	0.0296 (13)	0.0075 (12)	0.0116 (11)	−0.0013 (11)
C2	0.0402 (13)	0.0330 (13)	0.0317 (13)	0.0006 (11)	0.0086 (10)	−0.0027 (11)
C3	0.0489 (15)	0.0368 (14)	0.0342 (14)	0.0047 (12)	0.0048 (12)	0.0024 (11)
C4	0.0554 (17)	0.0370 (14)	0.0310 (13)	0.0016 (13)	0.0080 (12)	0.0008 (11)
C5	0.091 (3)	0.056 (2)	0.0337 (15)	0.0158 (18)	0.0123 (16)	0.0077 (14)
C6	0.075 (2)	0.0529 (19)	0.0468 (18)	0.0238 (17)	0.0095 (16)	0.0109 (15)
C11	0.0497 (16)	0.0382 (15)	0.0349 (13)	0.0018 (12)	0.0153 (12)	−0.0028 (11)
C12	0.0522 (17)	0.0479 (17)	0.0493 (17)	−0.0099 (14)	0.0195 (14)	−0.0114 (14)
C13	0.077 (2)	0.0550 (19)	0.0499 (19)	−0.0107 (17)	0.0214 (17)	−0.0184 (15)
C14	0.076 (2)	0.069 (2)	0.054 (2)	0.0061 (19)	0.0302 (17)	−0.0104 (17)
C15	0.064 (2)	0.088 (3)	0.070 (2)	−0.025 (2)	0.0379 (18)	−0.021 (2)
C16	0.061 (2)	0.069 (2)	0.064 (2)	−0.0229 (17)	0.0285 (16)	−0.0217 (18)
C21	0.0525 (16)	0.0441 (16)	0.0358 (14)	0.0111 (13)	0.0144 (12)	−0.0008 (12)
C22	0.0540 (19)	0.064 (2)	0.074 (2)	−0.0008 (17)	0.0196 (17)	−0.0130 (18)
C23	0.055 (2)	0.099 (3)	0.079 (3)	0.009 (2)	0.0224 (19)	−0.009 (2)
C24	0.078 (2)	0.094 (3)	0.054 (2)	0.038 (2)	0.0255 (18)	−0.002 (2)
C25	0.094 (3)	0.060 (2)	0.073 (2)	0.021 (2)	0.023 (2)	−0.0195 (19)
C26	0.067 (2)	0.0464 (18)	0.066 (2)	0.0080 (16)	0.0163 (17)	−0.0167 (16)
C30	0.023 (4)	0.040 (6)	0.038 (5)	−0.002 (4)	0.003 (3)	0.016 (4)
Cl1	0.090 (3)	0.102 (3)	0.186 (6)	−0.010 (2)	0.034 (3)	−0.072 (4)
Cl2	0.113 (3)	0.099 (4)	0.0580 (19)	0.0136 (19)	0.0249 (16)	0.0109 (16)
Cl3	0.048 (2)	0.087 (3)	0.136 (5)	−0.0110 (17)	0.004 (3)	−0.014 (2)
C31	0.120 (12)	0.095 (12)	0.069 (9)	0.058 (9)	0.015 (8)	−0.002 (8)
Cl1A	0.0494 (16)	0.0500 (17)	0.057 (2)	−0.0037 (10)	0.0128 (10)	−0.0085 (13)
Cl2A	0.143 (6)	0.285 (10)	0.057 (2)	0.049 (5)	0.021 (3)	0.062 (4)
Cl3A	0.110 (5)	0.048 (2)	0.126 (4)	−0.008 (2)	0.072 (4)	−0.0069 (18)

### Geometric parameters (Å, º)

**Table:**

S1—C1	1.666 (3)	C15—C16	1.542 (4)
S2—C1	1.657 (3)	C15—H15A	0.9800
N1—C2	1.345 (3)	C15—H15B	0.9800
N1—C4	1.392 (3)	C16—H16A	0.9800
N1—C21	1.482 (3)	C16—H16B	0.9800
N2—C2	1.329 (3)	C21—C26	1.499 (4)
N2—C3	1.400 (3)	C21—C22	1.504 (4)
N2—C11	1.487 (3)	C21—H21	0.9900
C1—C2	1.484 (4)	C22—C23	1.539 (5)
C3—C4	1.354 (4)	C22—H22A	0.9800
C3—C6	1.486 (4)	C22—H22B	0.9800
C4—C5	1.494 (4)	C23—C24	1.500 (6)
C5—H5A	0.9700	C23—H23A	0.9800
C5—H5B	0.9700	C23—H23B	0.9800
C5—H5C	0.9700	C24—C25	1.495 (6)
C6—H6A	0.9700	C24—H24A	0.9800
C6—H6B	0.9700	C24—H24B	0.9800
C6—H6C	0.9700	C25—C26	1.524 (5)
C11—C16	1.490 (4)	C25—H25A	0.9800
C11—C12	1.508 (4)	C25—H25B	0.9800
C11—H11	0.9900	C26—H26A	0.9800
C12—C13	1.521 (4)	C26—H26B	0.9800
C12—H12A	0.9800	C30—Cl1	1.579 (10)
C12—H12B	0.9800	C30—Cl3	1.744 (7)
C13—C14	1.485 (5)	C30—Cl2	1.807 (7)
C13—H13A	0.9800	C30—H30	0.9900
C13—H13B	0.9800	C31—Cl2A	1.605 (16)
C14—C15	1.509 (5)	C31—Cl3A	1.754 (15)
C14—H14A	0.9800	C31—Cl1A	1.896 (14)
C14—H14B	0.9800	C31—H31	0.9900
			
C2—N1—C4	108.3 (2)	C14—C15—H15B	109.5
C2—N1—C21	121.6 (2)	C16—C15—H15B	109.5
C4—N1—C21	129.8 (2)	H15A—C15—H15B	108.1
C2—N2—C3	108.7 (2)	C11—C16—C15	108.5 (3)
C2—N2—C11	121.8 (2)	C11—C16—H16A	110.0
C3—N2—C11	129.3 (2)	C15—C16—H16A	110.0
C2—C1—S2	115.8 (2)	C11—C16—H16B	110.0
C2—C1—S1	114.64 (19)	C15—C16—H16B	110.0
S2—C1—S1	129.54 (16)	H16A—C16—H16B	108.4
N2—C2—N1	108.8 (2)	N1—C21—C26	113.5 (2)
N2—C2—C1	125.6 (2)	N1—C21—C22	112.1 (2)
N1—C2—C1	125.6 (2)	C26—C21—C22	113.5 (2)
C4—C3—N2	106.8 (2)	N1—C21—H21	105.7
C4—C3—C6	128.6 (3)	C26—C21—H21	105.7
N2—C3—C6	124.6 (2)	C22—C21—H21	105.7
C3—C4—N1	107.3 (2)	C21—C22—C23	109.1 (3)
C3—C4—C5	128.4 (3)	C21—C22—H22A	109.9
N1—C4—C5	124.3 (3)	C23—C22—H22A	109.9
C4—C5—H5A	109.5	C21—C22—H22B	109.9
C4—C5—H5B	109.5	C23—C22—H22B	109.9
H5A—C5—H5B	109.5	H22A—C22—H22B	108.3
C4—C5—H5C	109.5	C24—C23—C22	111.9 (3)
H5A—C5—H5C	109.5	C24—C23—H23A	109.2
H5B—C5—H5C	109.5	C22—C23—H23A	109.2
C3—C6—H6A	109.5	C24—C23—H23B	109.2
C3—C6—H6B	109.5	C22—C23—H23B	109.2
H6A—C6—H6B	109.5	H23A—C23—H23B	107.9
C3—C6—H6C	109.5	C25—C24—C23	112.6 (3)
H6A—C6—H6C	109.5	C25—C24—H24A	109.1
H6B—C6—H6C	109.5	C23—C24—H24A	109.1
N2—C11—C16	113.9 (2)	C25—C24—H24B	109.1
N2—C11—C12	111.8 (2)	C23—C24—H24B	109.1
C16—C11—C12	113.3 (3)	H24A—C24—H24B	107.8
N2—C11—H11	105.7	C24—C25—C26	112.1 (3)
C16—C11—H11	105.7	C24—C25—H25A	109.2
C12—C11—H11	105.7	C26—C25—H25A	109.2
C11—C12—C13	110.8 (2)	C24—C25—H25B	109.2
C11—C12—H12A	109.5	C26—C25—H25B	109.2
C13—C12—H12A	109.5	H25A—C25—H25B	107.9
C11—C12—H12B	109.5	C21—C26—C25	109.1 (3)
C13—C12—H12B	109.5	C21—C26—H26A	109.9
H12A—C12—H12B	108.1	C25—C26—H26A	109.9
C14—C13—C12	112.2 (3)	C21—C26—H26B	109.9
C14—C13—H13A	109.2	C25—C26—H26B	109.9
C12—C13—H13A	109.2	H26A—C26—H26B	108.3
C14—C13—H13B	109.2	Cl1—C30—Cl3	114.7 (5)
C12—C13—H13B	109.2	Cl1—C30—Cl2	104.3 (4)
H13A—C13—H13B	107.9	Cl3—C30—Cl2	109.1 (4)
C13—C14—C15	111.8 (3)	Cl1—C30—H30	109.5
C13—C14—H14A	109.3	Cl3—C30—H30	109.5
C15—C14—H14A	109.3	Cl2—C30—H30	109.5
C13—C14—H14B	109.3	Cl2A—C31—Cl3A	112.4 (9)
C15—C14—H14B	109.3	Cl2A—C31—Cl1A	117.3 (10)
H14A—C14—H14B	107.9	Cl3A—C31—Cl1A	104.0 (7)
C14—C15—C16	110.9 (3)	Cl2A—C31—H31	107.6
C14—C15—H15A	109.5	Cl3A—C31—H31	107.6
C16—C15—H15A	109.5	Cl1A—C31—H31	107.6
			
C3—N2—C2—N1	−0.6 (3)	C2—N2—C11—C16	121.2 (3)
C11—N2—C2—N1	175.1 (2)	C3—N2—C11—C16	−64.0 (4)
C3—N2—C2—C1	177.6 (2)	C2—N2—C11—C12	−108.8 (3)
C11—N2—C2—C1	−6.7 (4)	C3—N2—C11—C12	66.0 (4)
C4—N1—C2—N2	0.4 (3)	N2—C11—C12—C13	174.7 (3)
C21—N1—C2—N2	−175.4 (2)	C16—C11—C12—C13	−54.9 (4)
C4—N1—C2—C1	−177.8 (2)	C11—C12—C13—C14	52.1 (4)
C21—N1—C2—C1	6.4 (4)	C12—C13—C14—C15	−54.1 (4)
S2—C1—C2—N2	87.5 (3)	C13—C14—C15—C16	56.8 (4)
S1—C1—C2—N2	−92.4 (3)	N2—C11—C16—C15	−173.7 (3)
S2—C1—C2—N1	−94.6 (3)	C12—C11—C16—C15	57.1 (4)
S1—C1—C2—N1	85.5 (3)	C14—C15—C16—C11	−57.2 (4)
C2—N2—C3—C4	0.5 (3)	C2—N1—C21—C26	−122.8 (3)
C11—N2—C3—C4	−174.8 (3)	C4—N1—C21—C26	62.4 (4)
C2—N2—C3—C6	−178.6 (3)	C2—N1—C21—C22	107.1 (3)
C11—N2—C3—C6	6.1 (5)	C4—N1—C21—C22	−67.7 (4)
N2—C3—C4—N1	−0.3 (3)	N1—C21—C22—C23	−173.0 (3)
C6—C3—C4—N1	178.8 (3)	C26—C21—C22—C23	56.9 (4)
N2—C3—C4—C5	−179.4 (3)	C21—C22—C23—C24	−53.3 (4)
C6—C3—C4—C5	−0.3 (5)	C22—C23—C24—C25	53.2 (5)
C2—N1—C4—C3	−0.1 (3)	C23—C24—C25—C26	−54.0 (5)
C21—N1—C4—C3	175.2 (3)	N1—C21—C26—C25	172.9 (3)
C2—N1—C4—C5	179.1 (3)	C22—C21—C26—C25	−57.6 (4)
C21—N1—C4—C5	−5.6 (5)	C24—C25—C26—C21	54.8 (4)

### Hydrogen-bond geometry (Å, º)

**Table:**

*D*—H···*A*	*D*—H	H···*A*	*D*···*A*	*D*—H···*A*
C15—H15*B*···S2^i^	0.98	2.76	3.650 (4)	151
C30—H30···S1^i^	0.99	2.79	3.646 (4)	145
C30—H30···S2^i^	0.99	2.68	3.543 (3)	145

Symmetry code: (i) *x*+1, *y*, *z*.
